# Radioulnar joint reconstruction: a novel surgical technique and biomechanical assessment for chronic instability

**DOI:** 10.1186/s40634-020-00291-1

**Published:** 2020-09-29

**Authors:** Gustavo Pacheco Martins Ferreira, Marcella Rodrigues Costa Simões, Haroldo Oliveira de Freitas Junior, Ubiratan Brum de Castro, Robinson Esteves Pires, Marco Antonio Percope de Andrade

**Affiliations:** 1 Hospital das Clínicas, Universidade Federal de Minas Gerais, Belo Horizonte, MG Brazil; 2 Serviço de Ortopedia e Traumatologia do Professor Matta Machado, Hospital da Baleia, Belo Horizonte, MG Brazil; 3 Serviço de Ortopedia e Traumatologia, Hospital Unimed-BH, Belo Horizonte, MG Brazil; 4 Departamento do Aparelho Locomotor, Universidade Federal de Minas Gerais, Belo Horizonte, MG Brazil

**Keywords:** Wrist joint, Wrist injuries, Triangular fibrocartilage joint instability, Orthopedic procedures

## Abstract

**Purpose:**

This study aims to describe and biomechanically evaluate a novel technique using a strip of the flexor carpi ulnaris tendon for distal radioulnar joint reconstruction.

**Methods:**

Surgical technique was thoroughly detailed, and a cadaveric biomechanical test was conducted to evaluate sagittal plane stability. Pronosupination range of motion was measured before and after the procedure. Dorsal and volar translation resistances were checked in three situations: with the uninjured triangular fibrocartilage complex, after its complete resection and after the surgical procedure.

**Results:**

For distal radioulnar joint translation, higher values were found both in dorsal and volar translation in situations with an injured triangular fibrocartilage complex, with means equal to 25.4 mm (SD: 9.4 mm) and 26.1 mm (SD: 8.0 mm), respectively. For intact triangular fibrocartilage complex, both dorsal and volar translations averages were 3.4 mm (SD: 0.9 mm) and 4.5 mm (SD: 1.8 mm), respectively. Finally, when evaluating dorsal and volar translations after surgical reconstruction, means were 6.3 mm (SD: 1.3 mm) and 6.8 mm (SD: 0.9 mm), respectively. Regarding supination and pronation range of motion, which ranged from 57.9 to 63.4^o^, there were no differences in mean and median measures, when the cadavers with intact and reconstructed triangular fibrocartilage complex were compared (*p* > 0.05).

**Conclusion:**

Promising mechanical evaluations encourage us to hypothesize that the technique effectively reconstructs the dorsal and volar radioulnar ligaments, preserving pronosupination and maintaining the physiological characteristics of the wrist joint. Clinical comparative studies are still necessary to fully validate this procedure.

**Level of evidence:**

Therapeutic - Level V.

## Background

The distal radioulnar joint is mainly stabilized by the triangular fibrocartilage complex (TFCC), which is composed by the homologous meniscus, the extensor carpi ulnaris tendon sheath, and by the volar and dorsal ulnocarpal and radioulnar ligaments [1, 2].

The distal radioulnar functions are tensioning the interosseous membrane to control the pronosupination movement, transferring load from carpus to forearm, and maintaining the anatomical relationship and stability between the radius and ulna [1, 3–5]. Therefore, distal radioulnar joint injuries may generate instability and consequently cause pain and functional impairment.

The most important associated factor to generate distal radioulnar joint injury is the distal radius fracture, especially when it occurs with the forearm in pronation [6]. Estimates reveal that 3–37% of these fracture patterns can concurrently damage the triangular fibrocartilage complex (TFCC) [7].

In an acute set of distal radioulnar joint reconstruction, the recommended treatment for subluxation management is joint fixation with forearm in supination or ligament repair through an open or arthroscopic procedure. In chronic injuries with arthritis, well-established surgical options include arthroplasty [8], bone resection [9], and arthrodesis [10]. In chronic injuries without arthritis, especially in patients with high functional demand, several treatment possibilities are currently available. However, a gold standard surgical procedure still lacks in the literature. In such cases, treatment aims to restore joint stability by using surrounding soft tissues as grafts. For distal radioulnar ligament reconstruction, most techniques generally use tendons passed through bone holes in the distal end of the radius and ulna [7, 11]. The smallest number of bone tunnels should be used to prevent iatrogenic fractures. Moreover, a free graft has the disadvantage of losing its vascularity while a tendon flap preserves circulatory viability and elastic properties [7].

Therefore, a ligament reconstruction which preserves the dynamic properties of this complex joint while adds a minimal surgical morbidity must be achieved whenever possible.

This study aims to describe and biomechanically test a novel surgical technique for chronic non-arthritic distal radioulnar joint instability, with the least number of bone tunnels as possible and using a vascularized flap of tendon.

## Methods

### Surgical technique

**Fig. 1:**
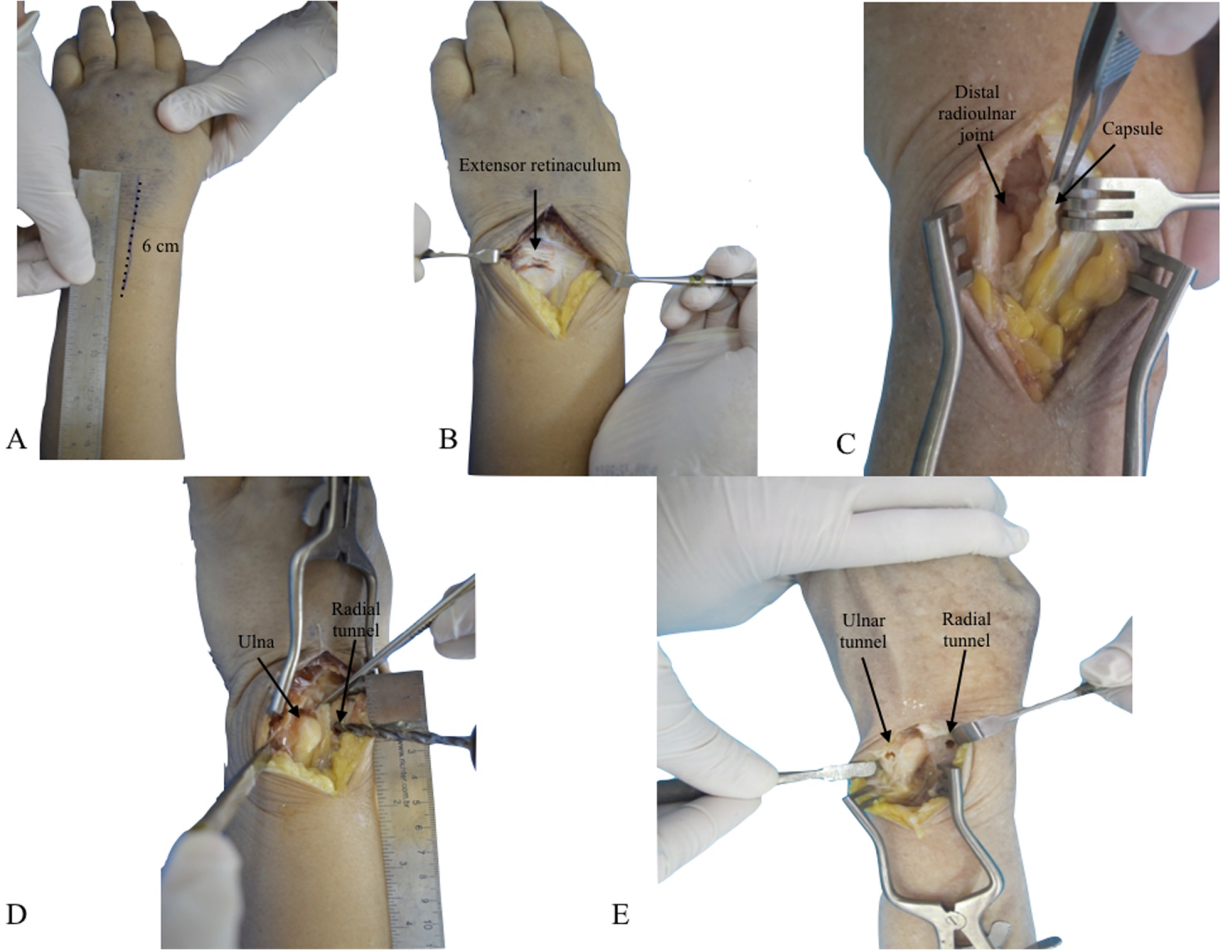
**a** Dorsal view of the wrist with the marked skin incision; (**b**) Dorsal view depicting the extensor retinaculum; (**c**) L-shaped capsulotomy with joint exposure; (**d**) Radial tunnel drilling; (**e**) Dorsal view of the wrist showing the radial and ulnar tunnels

**Fig. 2:**
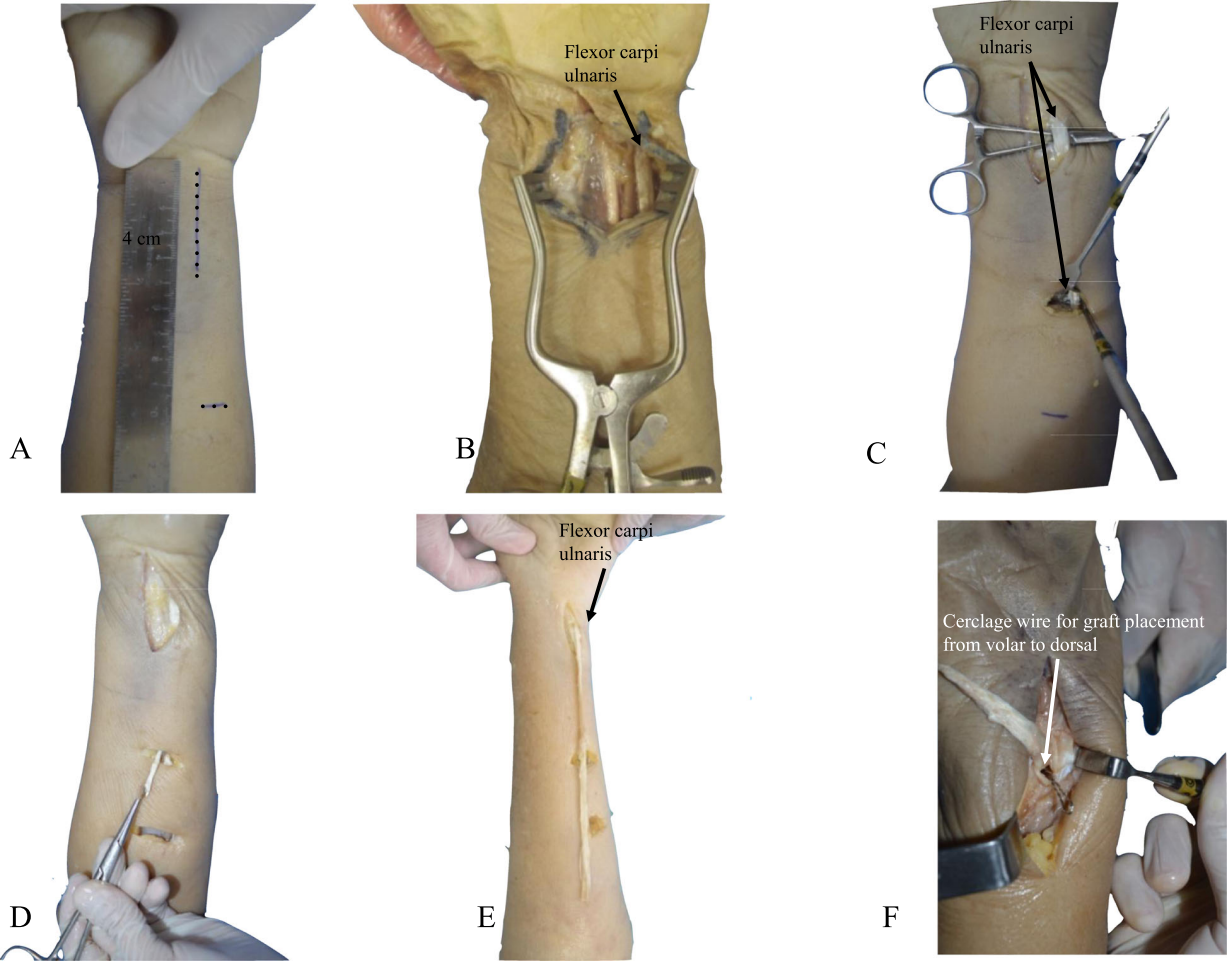
**a** Volar view of the wrist with the marked skin incision; (**b**) Volar approach showing the flexor carpi ulnaris; (**c**) Proximal and distal skin incisions with identification of the flexor carpi ulnaris; (**d**) Tendon strip release until the myotendinous junction; (**e**) Volar view of the wrist depicting the tendon strip graft; (**f**) Cerclage wire for graft placement from volar to dorsal

**Fig. 3:**
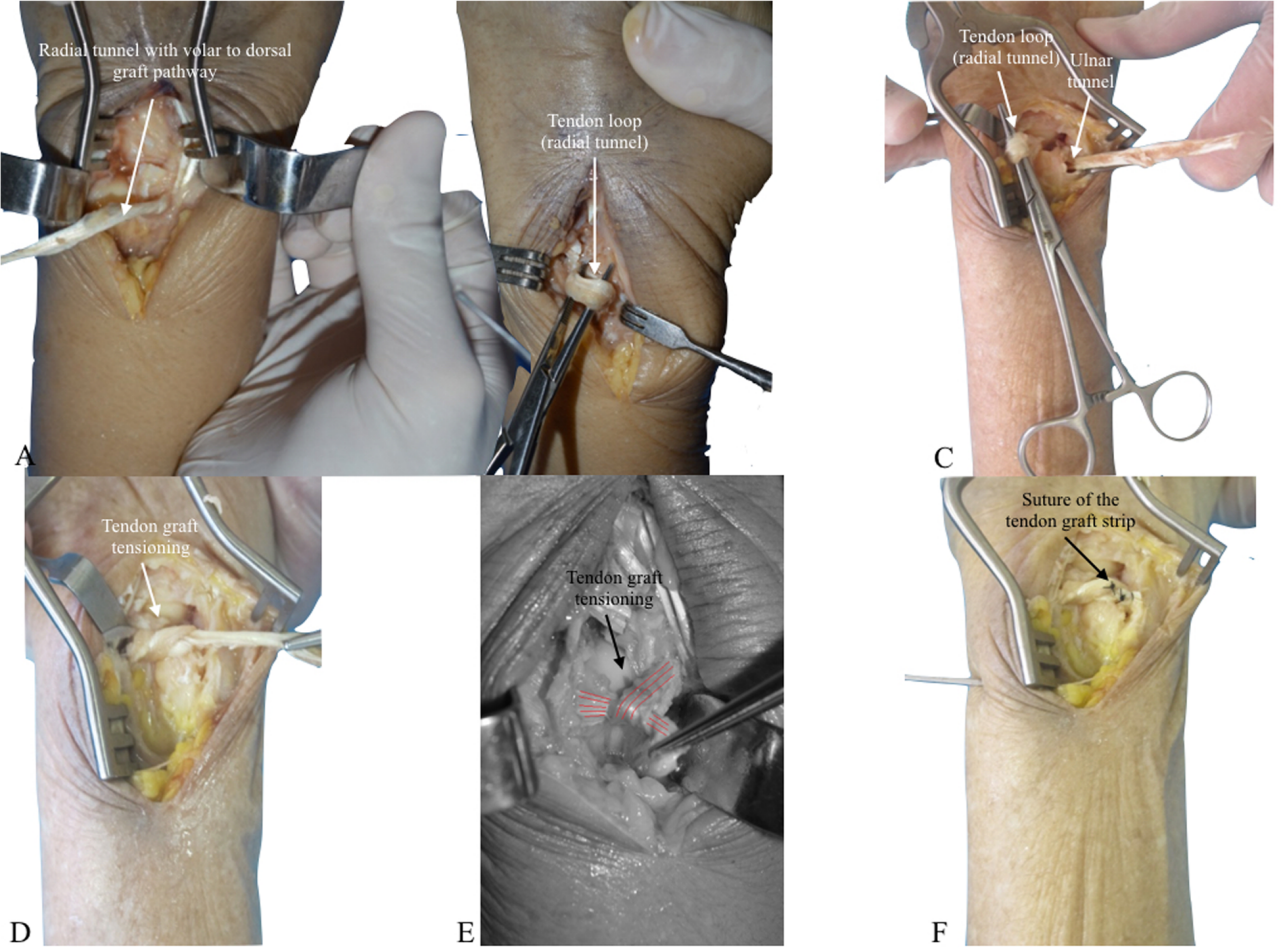
**a** Radial tunnel with volar to dorsal graft pathway; (**b**) Dorsal view of the wrist showing the dorsal tendon loop; (**c**) Dorsal view of the wrist showing the tendon placed through the ulnar tunnel; (**d**) Dorsal view showing tendon graft tensioning; (**e**) Red lines depicting tendon graft tensioning; (**f**) Suture of the tendon graft. Observe the K-wire for joint reduction maintenance

With the cadaver placed in supine position and the upper extremity supported on an accessory table, a 6 cm dorsal, longitudinal, and straight skin incision was performed at the level of the distal radioulnar joint (Fig. 1-a). Dissection was proceeded in planes, with exposure of the extensor retinaculum (Fig. 1-b), which was incised longitudinally, opening the fifth extensor compartment and the distal radioulnar joint capsule, with identification of the TFCC (Fig. 1-c). The sixth extensor compartment floor and the extensor carpi ulnaris tendon sheath were protected and maintained intact [4]. To simulate a radioulnar joint instability, TFCC structures are intentionally damaged with a scalpel. To be the nearest as possible of the anatomical origin of the radioulnar ligaments, a K-wire was inserted under fluoroscopy, to be sure of the correct positioning and then a bone tunnel was made with a 4.0 mm drill bit from a dorsal to volar direction at the ulnar aspect of the distal radius, 1.0 cm proximal to the articular surface and 1.0 cm radial to the sigmoid notch (Fig. 1 - d) [12]. Tunnel positioning must be precise, since the bone bridge left at the sigmoid notch margin is small and thin and creates a potential risk for iatrogenic fracture. Through the same approach, the ulna was reached, with identification and protection of the dorsal cutaneous branch of the ulnar nerve. The second bone tunnel was then made, with a 2.5 mm drill bit, at the base of the ulnar styloid process, also located 1.0 cm proximal to the articular surface (Fig. 1 - e). It was, as well, directed from dorsal to volar, searching to reproduce, as similar as possible, the ligament insertions. A 4 cm centrally longitudinal volar skin incision was then performed at the distal third of the forearm (Fig. 2 - a). After dissection and identification of anatomical structures, the flexor carpi ulnaris was isolated (Fig. 2 - b) For graft harvesting, two transverse approaches were performed on the volar aspect of the middle and proximal thirds of the forearm, over the path of the flexor carpi ulnaris tendon (Fig. 2 - c), splitting it longitudinally all over its length with a width of approximately 4 mm to resemble radioulnar ligament width [13] (Fig. 2 - e). The half strip was carefully cut proximally, close to myotendinous junction, taking care to leave the other half intact to preserve function (Fig. 2 - d). The distal part of the strip was kept attached and the graft was passed under the anatomical structures of the carpal tunnel to prevent local compression and then through the radial tunnel from the volar to dorsal direction, using a steel wire or tendon strainer to facilitate the procedure [2] (Figs. 2 - f and Fig. 3 - a). In the dorsal approach, the graft was passed back through the same radial tunnel, from dorsal to volar (Fig. 3 - b), maintaining a dorsal loop with a Kelly clamp inspired in the Bunnell technique. The graft was then passed from volar to dorsal through the ulnar tunnel (Fig. 3 - c), and in the dorsal approach, it was passed into the previously formed dorsal loop. The distal radioulnar joint was then reduced with the forearm in supination and the graft was tensioned, which led to reduction and stabilization in all planes (Figs. 3 - d and e). A 1.5 mm Kirschner wire, passed from ulnar to radial, 1 cm proximal to the bone tunnels, was used to fix the joint, helping maintenance of reduction.

After maximum manual tensioning, the distal end of the graft was sutured to itself with a 3.0 Nylon stitch, thereby achieving joint reduction and stability in both coronal and sagittal planes (Fig. 3 - f).

**Fig. 4:**
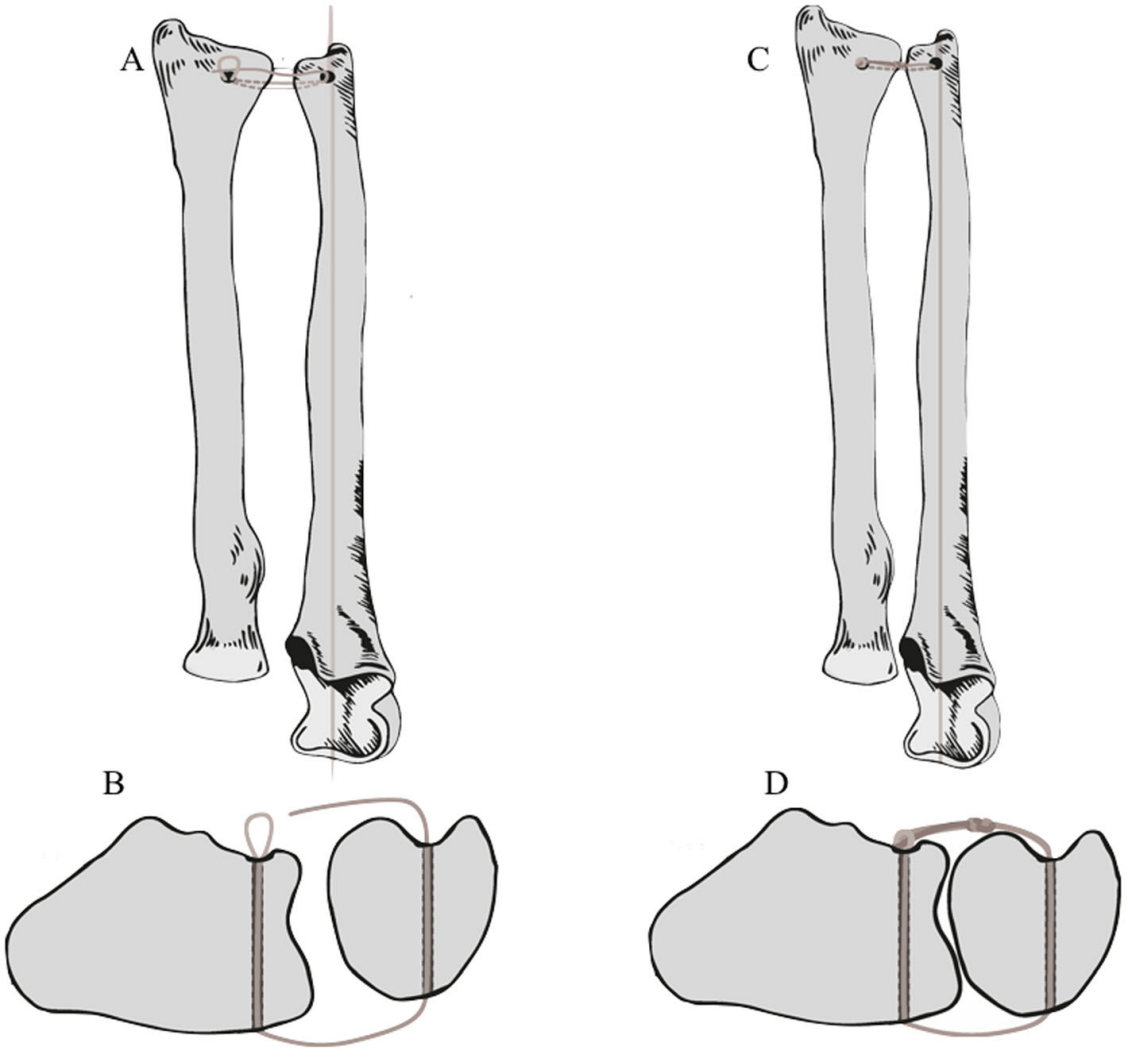
Technique illustration depicting the distal ulnar joint before (**a** and **b**) and after (**c** and **d**) tendon graft tensioning. Observe joint congruency and stability in both sagittal and coronal planes

Figure 4 depicts the surgical technique for distal radioulnar joint reconstruction (Fig. 4).

### Biomechanical assessment

Ten fresh frozen cadavers without signs of injuries or previous surgeries on the upper extremities were used to perform the presented surgical technique on a total of 20 wrists. Sample calculation was comparable to previous similar studies (Table 1). Mean and median of the number of forearms used in each of the five studies were calculated. Using these values, a minimum of 6.5 cadavers were required for biomechanical tests.

**Table 1:** Sample calculation according to previous studies

Article	Publication year	Journal	Sample details	Number of forearms
Ulnotriquetral augmentation tenodesis: A reconstructive procedure for dorsal subluxation of the distal radioulnar joint [14]	1982	The Journal of Hand Surgery	8 patients	8
Biomechanical evaluation of distal radioulnar reconstruction [15]	1993	The Journal of Hand Surgery	6 fresh cadaveric forearms	6
Reconstructive Procedure for Unstable Radial-Sided Triangular Fibrocartilage Complex Avulsions [16]	2005	The Journal of Hand Surgery	10 preserved cadaveric forearms	10
Comparison of Distal Radioulnar Joint Reconstructions Using an Active Joint Motion Simulator [1]	2005	The Journal of Hand Surgery	11 fresh cadaveric forearms	11
Palmar reconstruction of the triangular fibrocartilage complex for instability of the distal radioulnar joint: a biomechanical study [6]	2013	The Journal of Hand Surgery (European vol.)	7 fresh cadaveric forearms (elderly 71–93 years old)	7
**Mean (forearms)**				**8.5**
**Median (forearms)**				**6.5**

All procedures were performed by the same fellowship-trained, board-certified hand surgeon.

First, five cadavers were used to verify technique feasibility and reproducibility. After a step-by-step description of the surgical procedure, another cadaver was used as a pilot for mechanical test definition to measure dorsovolar translation resistance, range of movement before and after surgical technique, and flexor carpi ulnaris graft size. The final four cadavers, totaling eight reproductions, were used to perform biomechanical tests. Three of them were female and one was male, aged 29, 44, 67, and 77 years.

**Fig. 5:**
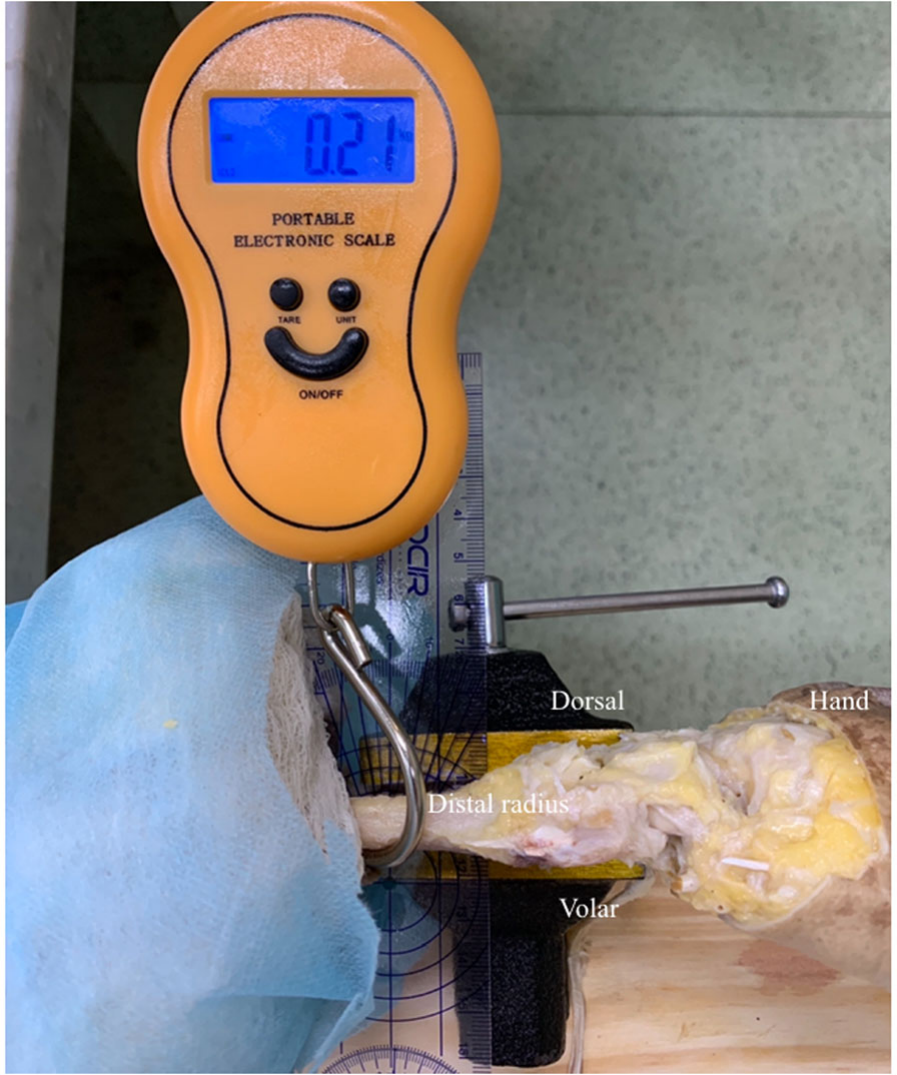
Dynamometer evaluating dorsal radius translation

Pronosupination range of motion was measured before and after the procedure using G-pro^R^ measurement software. The forearms were positioned on a table over a displacement measuring ruler with a device holding the ulna in neutral rotational positioning (Fig. 5). Dorsal and volar translation resistances were checked using an X-Tran^R^ electric dynamometer in three situations: with the uninjured triangular fibrocartilage complex, after its complete resection and after the surgical procedure. Dynamometer insertion point was determined in a bone tunnel created with a 4.0 mm drill bit at the radial metaphyseal transition, presenting no interference with described technique.

Traction force used was individualized for each forearm, and the chosen value was based on the maximum displacement measured with the integrity of the triangular fibrocartilage complex. Identical traction force was maintained with the resected TFCC and after performing the described technique.

## Results

After reproducing the described surgical steps in five cadavers, four additional cadavers were used to analyze pronosupination range of motion after the procedure as well as dorsal and volar stabilities.

Considering eight forearms in which tests were performed, the length of flexor carpi ulnaris strip used as a graft ranged from 111 to 155 mm (mean 132.9 mm, SD: 14.6 mm). Unused flexor carpi ulnaris strip length ranged from 0 to 34 mm (mean 13.0 mm, SD: 11.8 mm). Widths of the radius and ulna metaphyseal regions in the coronal plane were measured 1 cm proximal from their articular surfaces, at the region where the bone tunnels were performed. The radius width varied from 25 to 34.4 mm (mean 29.4 mm), and the ulna width varied from 16.3 to 24.6 mm (mean 19.2 mm). Forces applied to the eight forearms were 11, 15 and 16 kg (mean 14.5 and SD: 2.2 kg), which corresponded to the forces that caused the maximum displacement during the translation load, with the intact triangular fibrocartilage complex.

Table 2 depicts statistics regarding comparative characteristics. For distal radioulnar joint translation, higher values were found both in dorsal and volar translation in situations with an injured TFCC, with means equal to 25.4 mm (SD: 9.4 mm) and 26.1 mm (SD: 8.0 mm), respectively. For intact TFCC, both dorsal and volar translations averages were 3.4 mm (SD: 0.9 mm) and 4.5 mm (SD: 1.8 mm), respectively. Finally, when evaluating dorsal and volar translations after surgical reconstruction, means were 6.3 mm (SD: 1.3 mm) and 6.8 mm (SD: 0.9 mm), respectively (Table 2).

**Table 2:** Compared characteristics (translation and pronosupination)

Compared characteristics	Mean (SD)	***p*** value^*^	Median (IIQ)	***p*** value^**^	Min; Max
Dorsal translation – intact TFC (mm)	3.38 (0.9)	< 0.001	3 (1)	0.012	2; 5
Dorsal translation without TFC (mm)	25.38 (9.4)		23.5 (14)		14; 40
Mean difference (without TFC – with FCT)	22.00 (9.1)				
Dorsal post-surg translation (mm)	6.25 (1.3)	< 0.001	6 (2.5)	0.011	5; 8
Dorsal translation without TFC (mm)	25.38 (9.4)		23.5 (14)		14; 40
Mean difference (without TFC – post-surg)	19.13 (9.6)				
Dorsal translation – intact TFC (mm)	3.38 (0.9)	< 0.001	3 (1)	0.016	2; 5
Dorsal post-surg translation (mm)	6.25 (1.3)		6 (2.5)		5; 8
Mean difference (post-surg – with TFC)	2.87 (1.4)				
Volar translation – intact TFC (mm)	4.5 (1.8)	< 0.001	4 (2.5)	0.012	3; 8
Volar translation without TFC (mm)	26.13 (8.0)		25.5 (7)		12; 40
Mean difference (without TFC – with TFC)	21.63 (9.1)				
Volar translation post-surg (mm)	6.75 (0.9)	< 0.001	7 (0.5)	0.012	5; 8
Volar translation without TFC (mm)	26.13 (8.0)		25.5 (7)		12; 40
Mean difference (post-surg – without TFC)	19.38 (8.3)				
Volar translation – intact TFC (mm)	4.5 (1.8)	0.004	4 (2.5)	0.016	3; 8
Volar post-surg translation (mm)	6.75 (0.9)		7 (0.5)		5; 8
Mean difference (post-surg – intact TFC)	2.25 (1.5)				
Pre-surgical supination (degrees)	59.5 (13.0)	0.453	59 (24.5)	0.362	45; 76
Post-surgical supination (degrees)	57.9 (15.6)		53.5 (28.5)		39; 78
Mean difference (post-surg– pre-surg)	−1.6 (5.8)				
Pre-surgical pronation (degrees)	63.4 (6.7)	0.285	62.5 (7)	0.204	54; 76
Post-surgical pronation (degrees)	59.8 (8.7)		57 (15.5)		49; 72
Mean difference (post-surg– pre-surg)	−2.6 (6.4)				

*SD* Standard deviation.

*TFC* Triangular fibrocartilage complex.

Analyses of the medians indicated a similar behavior when comparing dorsal and volar translations.

Statistically significant differences existed when volar and dorsal translations were compared among wrists presenting intact TFCC, injured TFCC and after distal radioulnar ligament reconstruction (Table 2). However, differences between values of post-surgical translations and TFCC with integrity were significantly lower compared to the differences of values with injured or intact TFCC. Differences were, respectively, between 2 and 3 mm and between 19 and 22 mm (Table 2). Although not considered statistically similar, dorsal and volar translation values after applying the technique were very close to those of preserved anatomy, not being considered clinically relevant.

Regarding supination and pronation range of motion, which ranged from 57.9 to 63.4^o^, there were no differences in mean and median measures, when the cadavers with intact and reconstructed TFCC were compared (*p* > 0.05) (Table 2).

## Discussion

Distal radioulnar joint disorders are relatively common and may substantially impair forearm and wrist functioning. Some treatment options for chronic injuries remain controversial, mainly in a healthy joint without arthritis, and several surgical techniques are currently available.

Adams and Divelbiss classified treatment possibilities for chronic instability without arthritis in four groups: a) extra-articular radioulnar connection, b) indirect radioulnar connection with tenodesis or ulnocarpal tenoplasty, c) dynamic muscle transfer, and d) radioulnar ligament reconstruction, considered a gold standard treatment since it preserves pronosupination kinematics [17]. The novel technique presented in this study is included in the last abovementioned group. An ideal indication for the technique would be a young patient with no arthritis, presenting pain, weakness, and limited pronosupination range of motion due to chronic instability.

Compared to the previously described techniques for distal radiolunar reconstruction, this novel technique using a strip of the flexor carpi ulnaris tendon for joint reconstruction intend to present potential advantages: diminished iatrogenic risk of fracture, due to the two small tunnels with limited diameters; sufficient graft length for reconstruction, without need for any additional augmentation procedure; utilizing just one flap of the flexor carpi ulnaris, thereby preserving local vascularity and elastic properties of the graft when compared to a free graft [15]; preserving flexor carpi ulnaris function, since part of the tendon still remains intact keeping its origin and insertion; tunnel positioning and graft placement trying to mimetize the original ligament anatomy, addressing stability while maintaining normal range of pronosupination; preserving the extensor carpi ulnaris sheath and the sixth extensor compartment floor, important stabilizers of the distal radioulnar joint; and after graft tensioning, the graft is sutured into itself, conferring sagittal and coronal stabilization as demonstrated by biomechanical translation tests. It is noteworthy that, although no complications occurred during the cadaveric surgical procedures, some key points must be pointed out to optimize the technique: bone tunnels must be precise to prevent iatrogenic fractures; a steel wire or tendon strainer should be used to facilitate the passage of the tendons through the bone tunnels.

When the anteroposterior translation of the distal radioulnar joint, in cadavers with an intact TFCC and the ones with a reconstructed joint, was compared, difference does exist, even though small, which is expected for any technique of reconstruction. However, although this difference is very close to the preserved anatomy, one cannot disregard the potential risk of long-term osteoarthritis. What is important to show is the huge difference observed when cadavers with a ruptured TFCC were compared to the ones with the reconstructed ligament with the present technique, which confirms its efficacy.

The ideal reconstruction technique in any situation is the one that stabilizes the joint without restricting range or motion. In the particular case of the distal radioulnar joint, preservation of the pronosupination range of motion is one of the goals [18–20]. This novel technique did it, since there was no difference between the intact and the reconstructed joint, regarding range of motion.

Study limitations are related to the technique being performed on cadavers. Tests to measure reconstruction strength and tension were performed, but variations may occur since cadaver tissue presents diminished elasticity [6, 16]. In addition, tests on cadavers can lead to tissue fatigue after consecutive analyses [1, 15, 16]. To overcome this potential drawback and decrease fatigue effect in the present study, only one resistance test was used for each procedure. Finally, studies on cadavers disconsider physiological tissue healing.

A clinical study is currently being conducted by the authors to evaluate the safety and efficacy of this promising technique for anatomic radioulnar joint reconstruction in chronic instability without arthritis.

## Conclusion

This novel technique for distal radioulnar joint reconstruction using a strip of the flexor carpi ulnaris provides efficient stability in biomechanical volar and translation tests. Moreover, pronosupination range of motion is fully preserved and maintains the physiological characteristics of the wrist joint. Although clinical studies are still necessary to fully validate this procedure, our experimental findings are both promising and encouraging.

## Data Availability

The datasets used and/or analyzed during the current study are available from the corresponding author on reasonable request.

## References

[1] Gofton WT, Gordon KD, Dunning CE, Johnson JA, King GJW (2005). Comparison of distal radioulnar joint reconstructions using an active joint motion simulator. J Hand Surg Eur.

[2] Meyer D, Schweizer A, Nagy L (2017). Anatomic reconstruction of distal radioulnar ligaments with tendon graft for treating distal radioulnar joint instability. Tech Hand Upper Extrem Surg.

[3] Dy CJ, Ouellette EA, Makowski AL (2009). Extensor retinaculum capsulorrhaphy for ulnocarpal and distal radioulnar instability. Tech Hand Upper Extrem Surg.

[4] Hagert E, Ferreres A, Garcia-Elias M (2010). Nerve-sparing dorsal and volar approaches to the radiocarpal joint. J Hand Surg Am.

[5] Henry MH, Smith DW, Masson MV (2004). Reconstruction of distal radioulnar joint instability. J Am Soc Surg Hand.

[6] Kataoka T, Moritomo H, Omokawa S, Iida A, Wada T, Aoki M (2013). Palmar reconstruction of the triangular fibrocartilage complex for instability of the distal radioulnar joint: a biomechanical study. J Hand Surg Eur.

[7] Omokawa S, Lida A, Fujitani R, Onishi T, Tanaka Y (2014). Radiographic predictors of DRUJ instability with distal radius fractures. J Wrist Surg.

[8] Shecker LR, Bobb BA, Killion PE (2001). Distal ulnar prosthetic replacement. Orthop Clin North Am.

[9] Bowers WH (1985). Distal radioulnar joint arthroplasty: the hemiressection-interposition technique. J Hand Surg Am.

[10] Kapandji IA (1986). The Kapandji-sauve operation. Its techniques and indications in non-rheumatoid disease. Ann Chir Main.

[11] Bain GI, Bergman J (2007). The distal radioulnar joint. Tech Hand Upper Extrem Surg.

[12] Adams BD, Berger RA (2002). An anatomic reconstruction of the distal radioulnar ligament for posttraumatic distal radioulnar joint instability. J Hand Surg Am.

[13] Palmer AK, Werner FW (1981). The triangular fibrocartilage complex of the wrist: anatomy and function. J Hand Surg Am.

[14] Hui FC, Linscheid RL (1982). Ulnotriquetral augmentation tenodesis: a reconstructive procedure for dorsal subluxation of the distal radioulnar joint. J Hand Surg Am.

[15] Petersen MS, Adams BD (1993). Biomechanical evaluation of distal radioulnar reconstructions. J Hand Surg Am.

[16] Martineau PA, Bergeron S, Beckman L, Steffen T, Harvey EJ (2005). Reconstructive procedure for unstable radial-sided triangular fibrocartilage complex avulsions. J Hand Surg Am.

[17] Adams BD, Divelbiss BJ (2001). Reconstruction of the posttraumatic unstable distal radioulnar joint orthopedic. Clin North Am.

[18] Mesplié G, Grelet V, Léger O, Lemoine S, Ricarrère D, Geoffroy C (2017). Rehabilitation of distal radioulnar joint instability. Hand Surg Rehabil.

[19] Purisa H, Sezer I, Kabakas F, Tunçer S, Erturer E, Yazar M (2011). Ligament reconstruction using the Fulkerson-Watson method to treat chronic isolated distal radioulnar joint instability: short-term results. Acta Orthop Traumatol Turc.

[20] Zyluk A, Piotuch B (2013). Distal radioulnar joint instability: a review of literature. Pol Orthopaedic Traumatologic.

